# 

**Published:** 1898-12-31

**Authors:** 


					TQB HOSPITAL. " The standard of highest purity."?Laneet. iwsT, is?s.
CADBURY'S O ft** CADBURY'S
GOOOA oocoa
ABSOLUTELY PURE. NO DRUGS OR ZALKALIEg USED.
V\0!
I*icf?
S. C
'RWICH UOSRUTAJ,
ft. i ^VuLlSODIT WZSTKJIM LhlFlRMAJir
Wo-64(. Vol. XXY. [fK?~pS] Satubday,
Dec. 31st, 1898.
^^"NUaf.QLKAtilLJiUR niCH HOSB.UTAJ. .
[SubsoriptionTerms,] pBICE TWOPENCE.

				

